# Re-Infection Outcomes following One- and Two-Stage Surgical Revision of Infected Hip Prosthesis: A Systematic Review and Meta-Analysis

**DOI:** 10.1371/journal.pone.0139166

**Published:** 2015-09-25

**Authors:** Setor K. Kunutsor, Michael R. Whitehouse, Ashley W. Blom, Andrew D. Beswick, INFORM Team

**Affiliations:** Musculoskeletal Research Unit, School of Clinical Sciences, University of Bristol, Learning & Research Building (Level 1), Southmead Hospital, Southmead Road, Bristol, BS10 5NB, United Kingdom; Peking Union Medical College Hospital, CHINA

## Abstract

**Background:**

The two-stage revision strategy has been claimed as being the “gold standard” for treating prosthetic joint infection. The one-stage revision strategy remains an attractive alternative option; however, its effectiveness in comparison to the two-stage strategy remains uncertain.

**Objective:**

To compare the effectiveness of one- and two-stage revision strategies in treating prosthetic hip infection, using re-infection as an outcome.

**Design:**

Systematic review and meta-analysis.

**Data Sources:**

MEDLINE, EMBASE, Web of Science, Cochrane Library, manual search of bibliographies to March 2015, and email contact with investigators.

**Study Selection:**

Cohort studies (prospective or retrospective) conducted in generally unselected patients with prosthetic hip infection treated exclusively by one- or two-stage revision and with re-infection outcomes reported within two years of revision. No clinical trials were identified.

**Review Methods:**

Data were extracted by two independent investigators and a consensus was reached with involvement of a third. Rates of re-infection from 38 one-stage studies (2,536 participants) and 60 two-stage studies (3,288 participants) were aggregated using random-effect models after arcsine transformation, and were grouped by study and population level characteristics.

**Results:**

In one-stage studies, the rate (95% confidence intervals) of re-infection was 8.2% (6.0–10.8). The corresponding re-infection rate after two-stage revision was 7.9% (6.2–9.7). Re-infection rates remained generally similar when grouped by several study and population level characteristics. There was no strong evidence of publication bias among contributing studies.

**Conclusion:**

Evidence from aggregate published data suggest similar re-infection rates after one- or two-stage revision among unselected patients. More detailed analyses under a broader range of circumstances and exploration of other sources of heterogeneity will require collaborative pooling of individual participant data.

**Systematic Review Registration:**

PROSPERO 2015: CRD42015016559

## Introduction

Prosthetic joint infection (PJI) occurring within two years of hip replacement is mainly as a consequence of the surgical intervention[1] and is commonly associated with extreme pain, restricted movement, feelings of isolation, insecurity, and hopelessness, and may even lead to death [2, 3]. Several treatment options exist, but mainly involve one-stage or two-stage revision. The one-stage revision involves prosthesis removal, debridement, and re-implantation of a new prosthesis in one surgical procedure. In the two-stage revision, the removal of infected components and re-implantation of revision components are separated by a period of antibiotic therapy with no prosthesis in situ [4]. Of the two options, the two-stage revision strategy is used commonly and has been traditionally regarded as the “gold standard” [5]. Despite the opportunities for additional antimicrobial strategies[6] with the two-stage procedure, patients who undergo this procedure require extra hospital admission, undergo further major surgery, may experience considerable pain and disability during the period between operations and sometimes after the revision [7]. There is an increasing interest in the use of the single-stage revision as it may be associated with better patient reported outcomes; may reduce the requirement for prolonged stay in hospital; and overall healthcare costs of this procedure may be less. The best treatment option is currently uncertain.

There have been no randomised controlled trials comparing one- and two-stage revision procedures. However, several observational studies have assessed reinfection (re-infection and/or recurrence of infection) outcomes following the one-stage or two-stage surgical revisions and have reported inconsistent results. Gallo and colleagues reviewed 77 studies which included a total of 645 hips and reported overall re-infection rates after one- and two- stage hip prosthetic joint infection revision of 9.2% and 7.4% respectively. In their report, they concluded that one-stage revision was a less reliable approach for the primary outcome of recurrent infection [8]. In another review, Wolf and colleagues also reported an increased re-infection rate after one-stage compared to two-stage revision of infected total hip replacement [9]. Using a Markov cohort simulation decision analysis, they reported that the overall balance of risk and benefit favours the one-stage approach in the treatment of infection after a total hip replacement. Our group has also assessed re-infection outcomes of one- and two-stage revision of infected hip replacements using published studies that included populations mainly representative of patients in routine clinical practice [10]. We reported a lower re-infection rate at two years for one-stage revision (8.6%) compared to a two-stage revision (10.2%), though there was no significant difference in re-infection rates. The inconsistent evidence does not conclusively support a specific revision strategy for prosthetic hip infection. In addition, there were several features of these reviews (including ours) which limit the validity of the findings. First, the heterogeneous follow-up periods for re-infection outcomes by the studies included limited interpretation of the findings. Although our review assessed only re-infection outcomes within two years of revision surgery, this approach was not possible in all studies. Second, there was substantial heterogeneity among contributing studies which was not adequately explored. Third, publication bias was not assessed. Finally, none of the reviews conducted any assessment of re-infection outcomes following one- or two-stage revision across important study characteristics (e.g. geographical location, age at baseline, and surgical techniques). In addition, several relevant reports have been published since our previous review.

There is a requirement to find the best treatment option for PJI after hip replacement. Indeed, to compare the effectiveness of one-stage and two-stage revision strategies will require robust evidence from a carefully designed randomised clinical trial. With the low incidence of PJI (0.7% to 1.1% in Europe [11, 12] and 2.2% in the United States [13]) after total hip replacement, an appropriate definitive randomised trial with re-infection as the primary outcome may be unlikely in the short term. In the present study, we aimed to update our previous systematic review and meta-analysis by conducting more comprehensive analyses such as: (1) comprehensive quality assessment of relevant study domains such as prospective collection of data; endpoints appropriate to the aim of the study; unbiased assessment of the study endpoint; and follow-up period appropriate to the aim of the study, using a validated instrument employed in surgical studies; (2) comparing the effectiveness of the one- and two-stages under a range of study-level characteristics; (3) exploration of potential sources of heterogeneity between studies; and (4) assessment of publication bias.

## Methods

### Data sources and search strategy

We conducted this review using a predefined protocol, which has been registered in the PROSPERO prospective register of systematic reviews (CRD42015016559), and in accordance with PRISMA (Appendix A in S1 File) and MOOSE guidelines [14, 15](Appendix B in S1 File). We systematically searched for randomised controlled trials and cohort studies (both retrospective and prospective) reporting re-infection outcomes following one- or two-stage surgical revision of infected hip prosthesis in MEDLINE, EMBASE, Web of Science, and Cochrane register of controlled trials from March 2011 (date of our last search for the previous review) to March 2015. The computer-based searches combined free and medical subject headings and combination of key words related to hip replacement, infection, and revision with focus on one- and two-stage surgeries. There were no restrictions on language. Bibliographies of all retrieved articles and other relevant publications, including reviews and meta-analyses, were manually scanned for citations missed by the electronic search. Further details on the search strategy are presented in Appendix C in S1 File. No ethical approval was required for the conduct of this study.

### Eligibility criteria

Studies were included if they involved mainly unselected patients (i.e., patients representative of the general patient population) treated exclusively by one-stage or two-stage revision and had at least been followed up for two years after revision for re-infection (i.e., re-infection and/ or recurrence of infection). The term “re-infection” is henceforth used throughout the manuscript. We used a two-year follow-up period as PJIs occurring within two years of hip replacement are mainly as a consequence of the surgical intervention [1, 16]. Studies that reported case series of methods in selected group of patients (such as subsamples of patients who received revision in one- or two-stages or patients with a specific infection such as fungal infections); did not include patients with less than two years of follow-up; and studies with a sample size of < 10 were excluded from the review.

### Data extraction and assessment of methodological quality

The data extraction and quality assessment were conducted in duplicate by two independent reviewers (S.K.K., A.D.B.). A standardized predesigned data collection form was used for data extraction. Data were abstracted, where available, on study, publication date, geographical location, mean age, percentage of males, duration of follow-up after revision surgery, type of re-implantation used (cemented/cementless), whether a spacer was used, number of re-infection outcomes, and sample size. Each article was assessed using the inclusion criteria above and any disagreement regarding eligibility of an article was discussed, and agreement reached by consensus with a third reviewer (M.R.W). Authors of studies who had reported on one- or two-stage revisions but did not report the data we required were contacted to provide more information. Additionally, in the case of multiple publications, the study with the most up-to-date or comprehensive information was included. Methodological quality was assessed based on the Methodological Index for Non-Randomised Studies (MINORS), a validated instrument which is designed for assessment of methodological quality of non-randomised studies in surgery [17]. For non-comparative studies, it uses eight pre-defined domains namely: a clearly stated aim, inclusion of consecutive patients, prospective collection of data, endpoints appropriate to the aim of the study, unbiased assessment of the study endpoint, follow-up period appropriate to the aim of the study, loss to follow-up less than 5%, and prospective calculation of the study size. For each item, MINORS assigns 0 for not reported, 1 for reported but inadequate, or 2 for reported and adequate. The global ideal score is 16. The quality of the collective group of evidence was considered in accordance with the Grading of Recommendations Assessment, Development, Development, and Evaluation (GRADE) methodological quality criteria [18].

### Statistical analyses

The rate of re-infection (number of re-infections within two years of hip revision surgery/total number of participants) with 95% confidence intervals (CIs) was used as the primary outcome across studies. The rates were calculated using the Freeman-Tukey variance stabilising double arcsine transformation [19], because the use of inverse variance weight in fixed-effects meta-analysis is suboptimal when dealing with binary data with low rates. Using this method, the transformed rates are weighted slightly towards 50% and studies with zero rates are not excluded from the meta-analysis. Confidence intervals around these estimates were calculated using the Wilson method [20], since the asymptotic method produces intervals which may contain inadmissible values especially when the statistic is near the boundary [21, 22]. Summary rates of re-infection were pooled using random effects models to minimize the effect of between-study heterogeneity [23]. Statistical heterogeneity across studies was quantified using standard chi-square tests and the I^2^ statistic [24]. Study-level characteristics including geographical location, baseline age, type of reimplant used, use of a spacer, size of study, and study quality were pre-specified as characteristics for assessment of heterogeneity, which was conducted using stratified analysis and random effects meta-regression [25]. We assessed the potential for small study effects such as publication bias through formal tests, namely Egger’s linear regression test [26] and by comparing pooled results from studies involving ≥ 50 participants with those from smaller studies (< 50 participants). Begg’s funnel plots were not used as there is evidence to suggest that it is an inaccurate method for assessing publication bias in meta-analysis of proportion studies with low proportion outcomes [27]. All analyses were conducted using Stata version 13 (Stata Corp, College Station, Texas, USA). The dataset for our analyses can be found in S2 File.

## Results

### Study identification and selection

**Fig 1:**
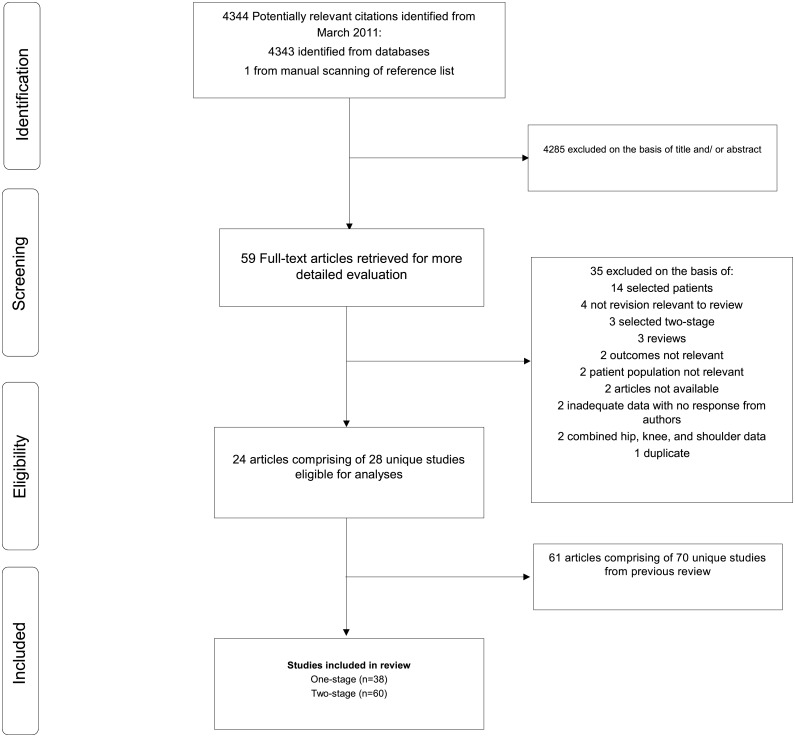
Selection of studies included in the meta-analysis.

Our initial search identified 4,343 potentially relevant citations plus one article identified manually from a reference list. After screening based on titles and abstracts, 59 articles remained for further evaluation. Following detailed assessments, 35 articles were excluded (Appendix D in S1 File). The remaining 24 articles (based on 28 unique studies), plus 61 relevant articles (based on 70 unique studies) from our previous review, met the inclusion criteria and were included in the meta-analysis (Fig 1; Table 1; Table A and Appendix E in S1 File). In aggregate, there were 98 unique studies (comprising of 5824 non-overlapping participants and 596 re-infection outcomes) included in the review.

**Table 1:** Summary characteristics of included studies.

	One-stage revision	Two-stage revision
**Eligible studies**		
Total number of studies included*	38	60
No. of studies identified for updated search*	7	21
**Participants**		
Total number of re-infections	290	306
Total number of participants	2536	3288
Median (IQR) age (years)	63.0 (64.6–69.0)	60.0 (64.0–69.0)
**Location**		
Europe	26 (2246)	32 (2012)
North America	8 (223)	16 (877)
South America	1 (32)	2 (68)
Asia	3 (35)	10 (331)
**Study characteristics**		
Median (IQR) follow-up (months)	35.0 (45.6–71.0)	35.0 (48.0–64.8)
Cemented reimplants^†^	15 (1385)	17 (778)
Use of spacers	-	29 (1579)
Interval between stages (months)	-	2.7 (4.5–5.7)

IQR = interquartile range; values are number of studies (number of participants) unless stated otherwise;

*, are not unique studies as there was overlap due to some studies reporting both revision strategies in the same article;

^†^, 16 and 21 studies in one- and two-stage revision studies respectively did not report the type of reimplantation used.

### Study characteristics and study quality

Table A in S1 File provides details and quality assessment scores of the eligible studies that assessed re-infection outcomes following one- or two-stage surgical revision. All included studies were longitudinal prospective or retrospective cohort studies carried out in North America (United States of America), South America (Brazil), Europe (United Kingdom, Ireland, Germany, France, Switzerland, Austria, Sweden, Spain, Italy, Finland, Greece, Poland, and Belgium), and Asia (South Korea, Japan, China, and Taiwan). No randomised controlled trials were identified. Methodological quality of included studies ranged from 9–16. The quality of the evidence (re-infection outcomes) based on GRADE criteria was very low for both one- and two-stage studies. For one-stage studies, median age for participants was 63 years and median follow-up duration was 35 months. The respective figures for two-stage studies were 60 years and 35 months (Table 1).

### One-stage revision

**Fig 2:**
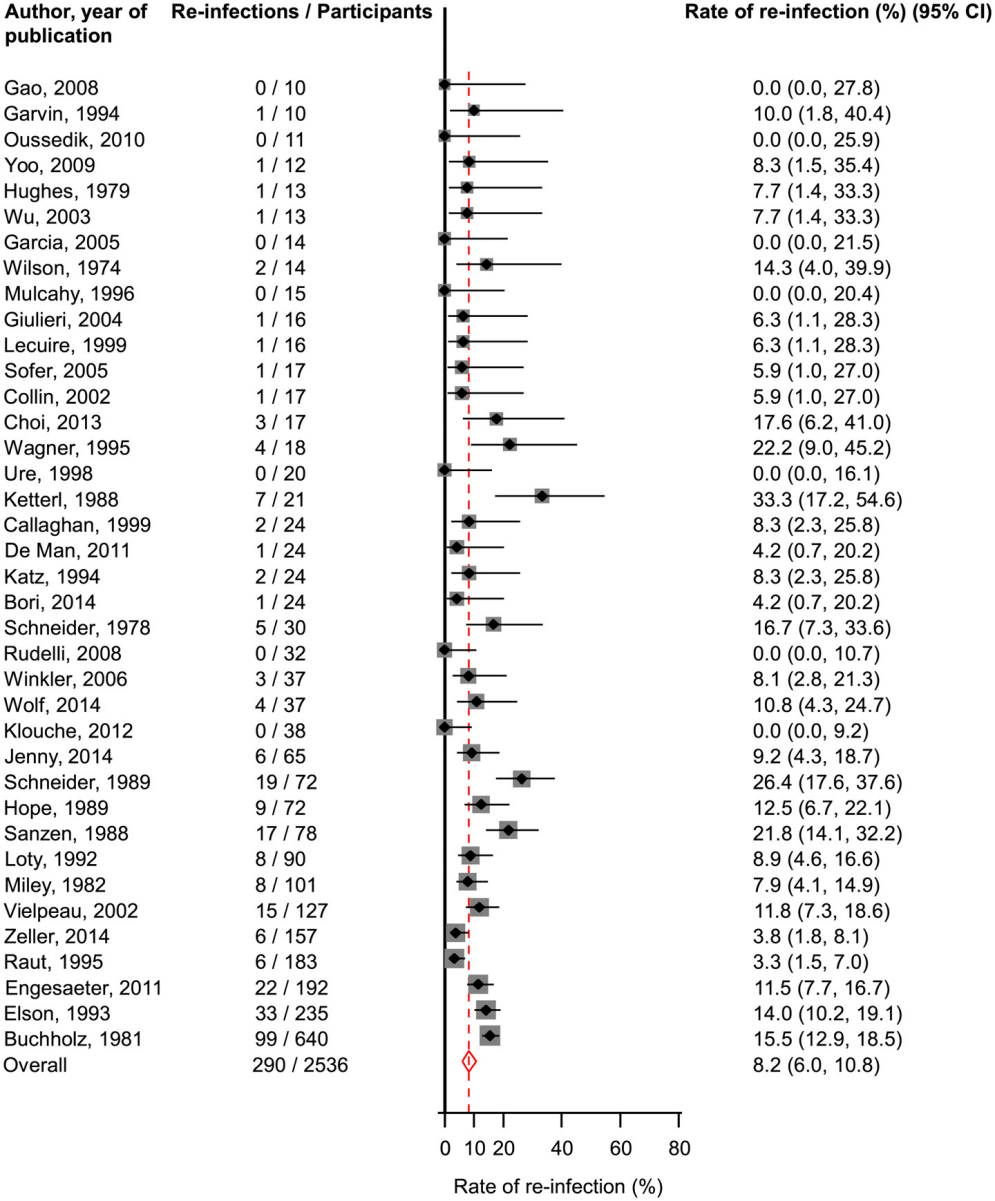
Rates of re-infection in unselected patients treated by one-stage revision. The summary estimates presented were calculated using random effects models; CI, confidence interval (bars).

Assessment of re-infection outcomes in unselected patients using the one-stage surgical revision strategy was reported in 38 studies involving 2536 participants. A total of 290 re-infection outcomes were reported (Table 1; Table A in S1 File). The pooled re-infection rate (95% CI) was 8.2% (6.0–10.8) (Fig 2). Excluding the single largest study comprising of 640 participants (with 99 re-infections) did not make any difference to the main finding 7.9% (5.6–10.5). There was evidence of moderate heterogeneity between contributing studies (*I* ^*2*^ = 66%, 52 to 76%; *P* < 0.001), which was not explained by any of the study level characteristics explored (*P* for meta-regression > 0.10; Fig A in S1 File). In further exploration of heterogeneity, this was substantially reduced, when we restricted the analysis to studies of the highest quality (quality score: 13–16). Among the remaining studies, the pooled re-infection rate (95% CI) was 7.0 (4.1–10.5), similar to the overall combined re-infection rate. Generally, similar rates of re-infection (overlapping confidence intervals) were observed across several subgroups. The Egger test for publication bias was not significant (*P* = 0.082), suggesting that studies with less striking results were not less likely to have been reported. In addition, we found no clear evidence of selective reporting when studies were grouped by size in meta-regression analysis (Fig A in S1 File).

### Two-stage revision

**Fig 3:**
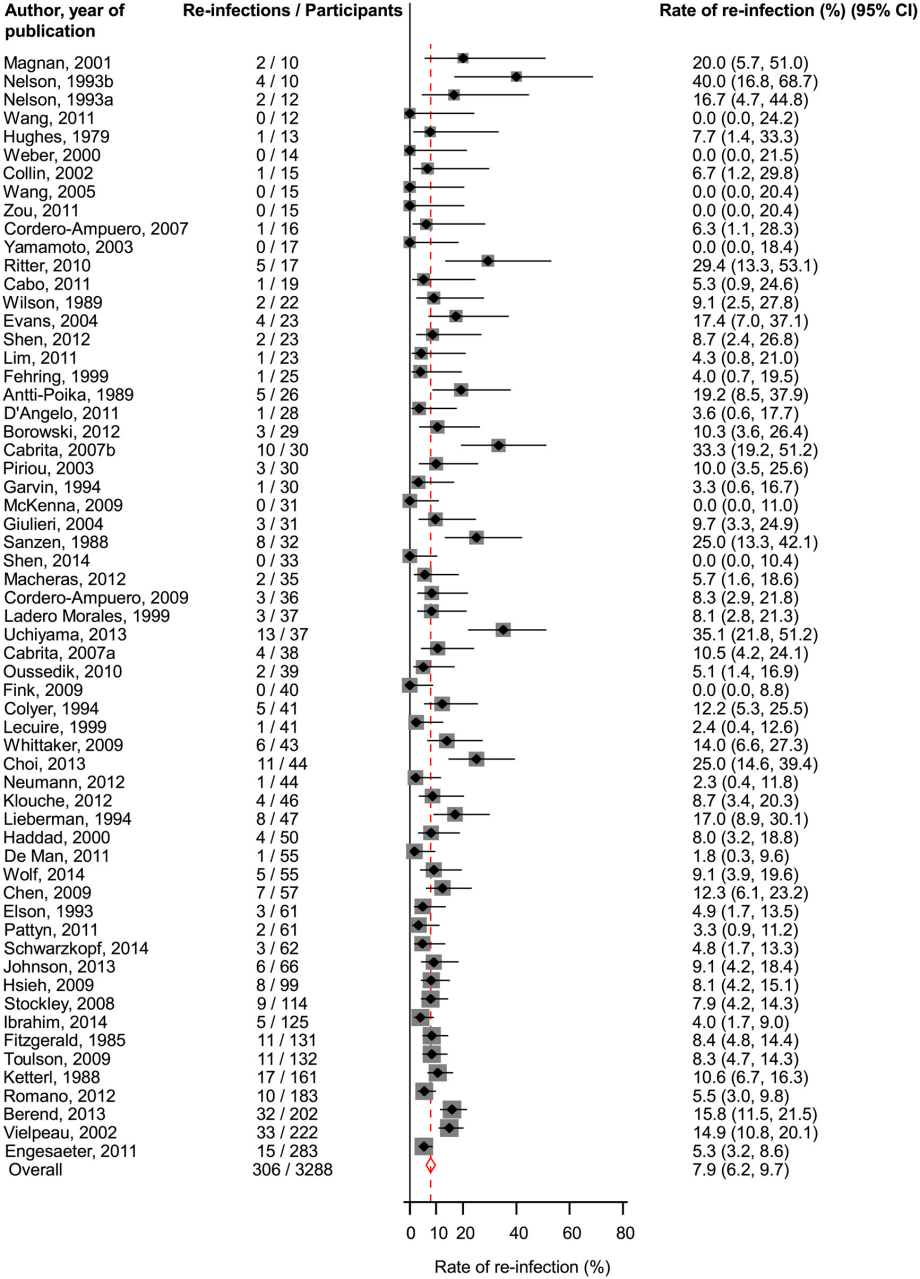
Rate of re-infection in unselected patients treated by two-stage revision. The summary estimates presented were calculated using random effects models; CI, confidence interval (bars).

Sixty studies comprising of 3288 participants (306 re-infections) reported on re-infection outcomes in unselected patients using the two-stage surgical revision strategy (Table 1; Table A in S1 File). The pooled re-infection rate (95% CI) was 7.9% (6.2–9.7) (Fig 3). We found evidence of moderate heterogeneity between contributing studies (*I* ^*2*^ = 60%, 48 to 70%; *P* < 0.001), which was not partly explained by any of the study characteristics assessed (*P* for meta-regression > 0.10; Fig B in S1 File). The rates of re-infection were generally similar across study relevant characteristics. There was no evidence of publication bias (Egger’s *P* = 0.649), consistent with the absence of selective reporting when studies were grouped by size in meta-regression analysis (Fig B in S1 File).

## Discussion

Findings of this updated meta-analysis generally concur and further extend that of previous reviews on the topic, providing several relevant findings that have not been previously addressed. This large-scale data indicate a re-infection rate of approximately 8% following one- or two-stage surgical revision for infected hip prosthesis in unselected patients. We confirmed the results of previous reviews[10, 28, 29] including ours [10], suggesting no significant superiority of either revision strategy over the other. Our results were however in contrast to two previous reviews [8, 9], which suggested significantly lower rates of re-infection following two-stage revision. Though the results of Wolf and colleagues [9] favoured the two-stage strategy in terms of effectively treating prosthetic hip infection, the one-stage strategy was reported as superior when other possible outcomes such as time interval required for treatment and death were also considered. The results of Gallo and colleagues [8] were also based on a total of only 645 hips for both revision strategies. Our findings showed that re-infection outcomes were generally consistent (albeit overlapping confidence intervals) across several study relevant characteristics for both revision strategies. Pooled analysis of one- or two-stage revision studies was characterised by moderate heterogeneity, which was not explained by any study level characteristic. The GRADE rating for the quality of the overall evidence was very low.

### Implications of findings

The choice of a superior revision strategy for prosthetic hip infection in unselected patients remains a difficult undertaking in light of the current findings. Despite this, the results are relevant and may have implications on surgical practice. Given that re-infection rate has traditionally been regarded as one of the most important factors or outcomes to determine the effectiveness of a revision strategy, our findings suggest that the two revision strategies may be comparable in terms of effectiveness in unselected patients. The two-stage revision has for several decades been presumed to be more effective that the one-stage for treating prosthetic joint infections [1, 30]. However, the two-stage strategy is not without its drawbacks. In addition to the significant functional impairment and increased mortality risk associated with this strategy [7, 9, 30], it has been estimated to cost much more than one-stage revision [31], given the potential of a longer hospitalisation period and an additional surgical procedure. The cost to the NHS of surgical revision of an infected hip replacement is estimated to be about £22,000 [32], with a two-stage costing about 70% more than a one-stage revision [31]. After the pioneering work of the one-stage strategy by Buchholz and colleagues [33], there has been an increasing interest in its use in recent years with routine adoption by many centres globally [34–36]. Although the one-stage strategy may require prolonged hospital admission to facilitate parenteral antibiotic therapy, it may be associated with better functional outcomes and huge cost savings [31, 35–37]. Whilst the pattern of our findings does not clearly yield supportive evidence for a specific revision strategy for prosthetic hip infection in unselected patients, it does highlight the importance of the one-stage strategy as a potential preferable strategy for surgeons, given the major merits of one major surgical procedure, reduced hospitalisation and functional impairment, and economic benefits.

### Strengths and limitations of the study

The strengths and potential limitations of this meta-analysis deserve consideration. The current study has some advantages with respect to the previous one. It is a comprehensive and updated assessment on the topic, with the inclusion of new studies and an update of previously reported studies; it therefore has enhanced power to compare the effectiveness of one- and two-stage revisions in terms of re-infection outcomes in greater detail. For example, meta-analysis of one-stage studies involved twice as many participants and re-infection outcomes than the previous review. We also conducted detailed analyses under a broad range of individual and study-level circumstances, which has previously not been done. As in the previous review [10], we only included studies that included consecutive and generally unselected patients with infection treated exclusively by one- or two-stage revision. Formal tests were unable to detect publication bias for studies in either revision strategy and there was no evidence of selective reporting when studies were grouped by size. Despite the lack of strong evidence of publication bias, it is not possible to discount completely the influence of selective reporting; since tests for publication bias have low statistical power.

There was only moderate evidence of heterogeneity among the contributing studies for either revision strategy, however, we systematically explored possible sources of heterogeneity using stratified analyses, meta-regression, and sensitivity analyses. Owing to the limited nature of the published data available, we could not explore re-infection outcomes by relevant subgroups such as co-morbidities (e.g., history of diabetes), type of fixation (cemented or uncemented), and use of antibiotics in cement or spacer. Although the aim was to include only studies that involved unselected patients, we recognise an earlier phase of selection related to management without further replacement in some studies. A detailed assessment of the definition of re-infection could not be undertaken as this was not clearly reported by majority of studies. We however conducted a quality assessment using a validated instrument for non-randomised surgical studies, to provide an unbiased assessment of re-infection outcomes among 7 other pre-defined domains. We used only data on re-infection within two years of revision surgery, which was sometimes confirmed by contacting the study authors concerned. However, it is possible some re-infection outcomes may have occurred after two years, given that not all authors responded to our request for additional information. Despite the comprehensive analysis and results, the findings should be interpreted in context of the limitations available. Given the limitations of aggregate published data, the very low quality rating of the overall evidence, and in the absence of robust evidence from a carefully designed randomised clinical trial, we propose a worldwide collaborative meta-analysis of individual participant data (IPD) from relevant studies. Within our Infection Orthopaedic Management (INFORM) Programme, which is involved in developing and establishing optimum management strategies for prosthetic joint infections within the NHS [38], work is currently underway to conduct a worldwide collaborative meta-analysis of IPD to address the existing uncertainties. Our study protocol has already been published.[39] Compared with only aggregated published data, access to individual-level from each prospective study contributing to this collaboration should enable: i) a consistent approach to the definition of the primary outcome (two-year incidence of re-infection); ii) a common approach across studies to statistical analyses and a consistent approach to adjustment for potential confounders; iii) greater ability to explore and identify sources of between-study heterogeneity; iv) ability to assess secondary outcomes such as function, pain, and death; and v) inclusion in the analysis of key prospective studies worldwide should help avoid biases due to selective inclusion of studies and enhance the generalisability of the study results. Relevant investigations in the INFORM Programme should help provide a more robust assessment on whether the one-stage is an equivalent or better revision strategy for prosthetic hip infection compared to the two-stage.

## Conclusions

In conclusion, evidence from aggregate published data suggest a re-infection rate of approximately 8% after one- or two-stage revision among unselected patients. More detailed analyses under a broader range of circumstances and exploration of other sources of heterogeneity will require collaborative pooling of individual participant data.

## Supporting Information

S1 File (DOC)

S2 File (CSV)
