# Supplementary material for: Re-Infection Outcomes following One- and Two-Stage Surgical Revision of Infected Hip Prosthesis: A Systematic Review and Meta-Analysis
Source: PLoS One. 2015 Sep 25;10(9):e0139166. doi: 10.1371/journal.pone.0139166 (PMC4583275; doi:10.1371/journal.pone.0139166)
Supplement: S1 File — (DOC) [file pone.0139166.s001.doc]

**SUPPLEMENTARY MATERIAL**

| **Appendix A** | PRISMA checklist |
| --- | --- |
| **Appendix B** | MOOSE checklist |
| **Appendix C** | Literature search strategy |
| **Appendix D** | Reference list of excluded studies |
| **Appendix E** | Reference list of studies included in review |
| **Table A** | Characteristics of prospective studies included in review |
| **Figure A** | Rates of re-infection in unselected patients treated by one-stage revision, grouped according to study and population level characteristics |
| **Figure B** | Rates of re-infection in unselected patients treated by two-stage revision, grouped according to study and population level characteristics |

**Appendix A.** PRISMA checklist

| **Section/topic** | **Item No** | **Checklist item** | **Reported on page No** |
| --- | --- | --- | --- |
| **Title** | | | |
| Title | 1 | Identify the report as a systematic review, meta-analysis, or both | 1 |
| **Abstract** | | | |
| Structured summary | 2 | Provide a structured summary including, as applicable, background, objectives, data sources, study eligibility criteria, participants, interventions, study appraisal and synthesis methods, results, limitations, conclusions and implications of key findings, systematic review registration number | 2 |
| **Introduction** | | | |
| Rationale | 3 | Describe the rationale for the review in the context of what is already known | 4-5 |
| Objectives | 4 | Provide an explicit statement of questions being addressed with reference to participants, interventions, comparisons, outcomes, and study design (PICOS) | 5 |
| **Methods** | | | |
| Protocol and registration | 5 | Indicate if a review protocol exists, if and where it can be accessed (such as web address), and, if available, provide registration information including registration number | 2 |
| Eligibility criteria | 6 | Specify study characteristics (such as PICOS, length of follow-up) and report characteristics (such as years considered, language, publication status) used as criteria for eligibility, giving rationale | 6 |
| Information sources | 7 | Describe all information sources (such as databases with dates of coverage, contact with study authors to identify additional studies) in the search and date last searched | 6 |
| Search | 8 | Present full electronic search strategy for at least one database, including any limits used, such that it could be repeated | Appendix C |
| Study selection | 9 | State the process for selecting studies (that is, screening, eligibility, included in systematic review, and, if applicable, included in the meta-analysis) | 6-7 |
| Data collection process | 10 | Describe method of data extraction from reports (such as piloted forms, independently, in duplicate) and any processes for obtaining and confirming data from investigators | 6-7 |
| Data items | 11 | List and define all variables for which data were sought (such as PICOS, funding sources) and any assumptions and simplifications made | 6-7 |
| Risk of bias in individual studies | 12 | Describe methods used for assessing risk of bias of individual studies (including specification of whether this was done at the study or outcome level), and how this information is to be used in any data synthesis | 7-8 |
| Summary measures | 13 | State the principal summary measures (such as risk ratio, difference in means). | 7-8 |
| Synthesis of results | 14 | Describe the methods of handling data and combining results of studies, if done, including measures of consistency (such as I^2^ statistic) for each meta-analysis | 7-8 |
| Risk of bias across studies | 15 | Specify any assessment of risk of bias that may affect the cumulative evidence (such as publication bias, selective reporting within studies) | 7-8 |
| Additional analyses | 16 | Describe methods of additional analyses (such as sensitivity or subgroup analyses, meta-regression), if done, indicating which were pre-specified | 7-8 |
| **Results** | | | |
| Study selection | 17 | Give numbers of studies screened, assessed for eligibility, and included in the review, with reasons for exclusions at each stage, ideally with a flow diagram | 8 and Fig. 1 |
| Study characteristics | 18 | For each study, present characteristics for which data were extracted (such as study size, PICOS, follow-up period) and provide the citations | 8-9, Table A |
| Risk of bias within studies | 19 | Present data on risk of bias of each study and, if available, any outcome-level assessment (see item 12). | 9-10, Table A |
| Results of individual studies | 20 | For all outcomes considered (benefits or harms), present for each study (a) simple summary data for each intervention group and (b) effect estimates and confidence intervals, ideally with a forest plot | 9-10, Figs. 2-3 |
| Synthesis of results | 21 | Present results of each meta-analysis done, including confidence intervals and measures of consistency | 9-10, Figs. 2-3 |
| Risk of bias across studies | 22 | Present results of any assessment of risk of bias across studies (see item 15) | 9-10 |
| Additional analysis | 23 | Give results of additional analyses, if done (such as sensitivity or subgroup analyses, meta-regression) (see item 16) | 9-10, Figure A; Figure A |
| **Discussion** | | | |
| Summary of evidence | 24 | Summarise the main findings including the strength of evidence for each main outcome; consider their relevance to key groups (such as health care providers, users, and policy makers) | 10-11 |
| Limitations | 25 | Discuss limitations at study and outcome level (such as risk of bias), and at review level (such as incomplete retrieval of identified research, reporting bias) | 11-12 |
| Conclusions | 26 | Provide a general interpretation of the results in the context of other evidence, and implications for future research | 12 |
| **Funding** | | | |
| Funding | 27 | Describe sources of funding for the systematic review and other support (such as supply of data) and role of funders for the systematic review | 13 |

**Appendix B.** MOOSE checklist

**Re-infection outcomes following one- and two-stage surgical revision of infected hip prosthesis: A systematic review and meta-analysis**

| **Criteria** | | **Brief description of how the criteria were handled in the review** |
| --- | --- | --- |
| **Reporting of background** | |  |
| √ | Problem definition | Prosthetic joint infection occurring within two years of hip replacement is mainly as a consequence of the surgical intervention. Two main treatment options exist and which include one-stage or two-stage revision. Several studies and reviews have been carried out on this topic, but the best treatment option is currently uncertain. In this context, we have carried out an updated systematic review and meta-analysis of longitudinal studies to compare the effectiveness of the one- and two-stage revision strategies in terms of re-infection outcomes |
| √ | Hypothesis statement | There is no difference in re-infection outcomes after one-stage or two-stage revision surgery for prosthetic hip joint infection |
| √ | Description of study outcomes | Rates of re-infection (number of re-infections within two years of hip revision surgery/total number of participants |
| √ | Type of exposure | One-stage and two-stage surgical revision of infected hip prosthesis |
| √ | Type of study designs used | Longitudinal studies (retrospective, prospective, or randomised controlled trials) |
| √ | Study population | Unselected patients |
| **Reporting of search strategy should include** | |  |
| √ | Qualifications of searchers | Setor Kunutsor, PhD; Andrew Beswick, BSc |
| √ | Search strategy, including time period included in the synthesis and keywords | Time period: from March, 2011 (date of our last search for the previous review) to March, 2015.  The detailed search strategy can be found in Appendix 3. |
| √ | Databases and registries searched | MEDLINE, EMBASE, Web of Science, and Cochrane databases |
| √ | Search software used, name and version, including special features | OvidSP was used to search EMBASE and MEDLINE  EndNote used to manage references |
| √ | Use of hand searching | We searched bibliographies of retrieved papers |
| √ | List of citations located and those excluded, including justifications | Details of the literature search process are outlined in the flow chart. The citation list for excluded studies is in Appendix 5. |
| √ | Method of addressing articles published in languages other than English | We placed no restrictions on language |
| √ | Method of handling abstracts and unpublished studies | We contacted several investigators for unpublished data and abstracts on the associations |
| √ | Description of any contact with authors | We contacted authors of studies that did not provide adequate data for analysis |
| **Reporting of methods should include** | |  |
| √ | Description of relevance or appropriateness of studies assembled for assessing the hypothesis to be tested | Detailed inclusion and exclusion criteria are described in the Methods section. |
| √ | Rationale for the selection and coding of data | Data extracted from each of the studies were relevant to the population characteristics, study design, exposure, and outcome. |
| √ | Assessment of confounding | We included only studies where populations were unselected |
| √ | Assessment of study quality, including blinding of quality assessors; stratification or regression on possible predictors of study results | Study quality was assessed based on the Methodological Index for Non-Randomised Studies (MINORS), a validated instrument which is designed for assessment of methodological quality of non-randomised studies in surgery |
| √ | Assessment of heterogeneity | Heterogeneity of the studies was quantified with I^2^ statistic that provides the relative amount of variance of the summary effect due to the between-study heterogeneity and explored using meta-regression and stratified analyses |
| √ | Description of statistical methods in sufficient detail to be replicated | Description of methods of meta-analyses, sensitivity analyses, meta-regression and assessment of publication bias are detailed in the methods. We performed random effects meta-analysis with Stata 13. |
| √ | Provision of appropriate tables and graphics | Table 1 and Table A; Figs. 1-3; Figure A; Figure B |
| **Reporting of results should include** | |  |
| √ | Graph summarizing individual study estimates and overall estimate | Figs. 2-3 |
| √ | Table giving descriptive information for each study included | Table 1 and Table A |
| √ | Results of sensitivity testing | Sensitivity analysis was conducted to assess the influence of some large studies and low quality studies on the pooled estimate. This was done by omitting such studies and calculating a pooled estimate for the remainder of the studies |
| √ | Indication of statistical uncertainty of findings | 95% confidence intervals were presented with all summary estimates, I^2^ values and results of sensitivity analyses |
| **Reporting of discussion should include** | |  |
| √ | Quantitative assessment of bias | Sensitivity analyses indicate heterogeneity in strengths of the association due to most common biases in observational studies. The systematic review is limited in scope, as it involves published data. Individual participant data is needed. Limitations have been discussed. |
| √ | Justification for exclusion | All studies were excluded based on the pre-defined inclusion criteria in methods section. |
| √ | Assessment of quality of included studies | Brief discussion included in ‘Methods’ section |
| **Reporting of conclusions should include** | |  |
| √ | Consideration of alternative explanations for observed results | Discussion |
| √ | Generalization of the conclusions | Discussed in the context of the results. |
| √ | Guidelines for future research | We recommend analyses of individual participant data |
| √ | Disclosure of funding source | In “Acknowledgement” section |

**Appendix C.** Literature search strategy

Relevant studies, published from March, 2011 (date of our last search for the previous review) to March, 2015 (date last searched), were identified through electronic searches not limited to the English language using MEDLINE, EMBASE, Web of Science, and Cochrane databases. Electronic searches were supplemented by scanning reference lists of articles identified for all relevant studies (including review articles), by hand searching of relevant journals and by correspondence with study investigators. The computer-based searches combined search terms related to hip replacement, infection, and revision with focus on one- and two stage surgeries without language restriction.

1 exp Prosthesis-Related Infections/ or prosthesis-related infection*.mp. or exp Sepsis/ (101971)

2 exp Infection/ or exp Wound Infection/ or exp Surgical Wound Infection/ or infection*.mp. (1603015)

3 wound infection.mp. or exp Wound Infection/ (44687)

4 arthroplasty.mp. or exp Arthroplasty, Replacement/ or exp Arthroplasty/ or exp Arthroplasty, Replacement, Hip/ (52003)

5 exp Arthroplasty, Replacement, Hip/ or exp Arthroplasty, Replacement/ or Replacement.mp. (204405)

6 exp Hip/ or exp Arthroplasty, Replacement, Hip/ or hip.mp. (107123)

7 exp Hip Prosthesis/ or exp Arthroplasty, Replacement, Hip/ or hip replacement.mp. (31655)

8 exp Hip Prosthesis/ or total hip.mp. or exp Arthroplasty, Replacement, Hip/ (34732)

9 hip arthroplasty.mp. (12045)

10 total hip replacement.mp. or exp Arthroplasty, Replacement, Hip/ (20706)

11 exp Arthroplasty, Replacement, Hip/ or total hip arthroplasty.mp. (20637)

12 exp Arthroplasty, Replacement, Hip/ or exp Hip Prosthesis/ or hip prosthes*.mp. (30397)

13 1-stage.mp. (1576)

14 2-stage.mp. (2828)

15 one stage.mp. (8705)

16 two stage.mp. (15977)

17 one-stage.mp. (8705)

18 two-stage.mp. (15977)

19 single stage.mp. (4349)

20 single-stage.mp. (4349)

21 prosthesis exchange.mp. (15)

22 direct exchange.mp. (153)

23 direct-exchange.mp. (153)

24 Arthroplasty, Replacement, Hip/ or revision arthroplasty.mp. (18017)

25 exp Arthroplasty, Replacement, Hip/ or staged revision.mp. (17490)

26 reoperation.mp. or exp Reoperation/ (77278)

27 reimplantation.mp. or exp Replantation/ (10449)

28 1 or 2 or 3 (1606087)

29 4 or 5 or 6 or 7 or 8 or 9 or 10 or 11 or 12 (295528)

30 13 or 14 or 15 or 16 or 17 or 18 or 19 or 20 or 21 or 22 or 23 or 24 or 25 or 26 or 27 (129343)

31 28 and 29 and 30 (5795)

32 limit 31 to yr="2011 -Current" (1602)

Each part was specifically translated for searching the other databases (EMBASE, Web of Science, and Cochrane databases)

**Appendix D.** Reference list of excluded studies

1. Hansen E, Tetreault M, Zmistowski B, et al. Outcome of one-stage cementless exchange for acute postoperative periprosthetic hip infection. *Clinical Orthopaedics & Related Research.* 2013;471(10):3214-3222.

2. Degen RM, Davey JR, Davey JR, Howard JL, McCalden RW, Naudie DD. Does a prefabricated gentamicin-impregnated, load-bearing spacer control periprosthetic hip infection? *Clinical Orthopaedics & Related Research.* 2012;470(10):2724-2729.

3. Huang R, Hu CC, Adeli B, Mortazavi J, Parvizi J. Culture-negative periprosthetic joint infection does not preclude infection control. *Clinical Orthopaedics & Related Research.* 2012;470(10):2717-2723.

4. Webb JE, Schleck CD, Larson DR, Lewallen DG, Trousdale RT. Mortality of elderly patients after two-stage reimplantation for total joint infection: a case-control study. *J Arthroplasty.* 2014;29(11):2206-2210.

5. Tsung JD, Rohrsheim JAL, Whitehouse SL, Wilson MJ, Howell JR. Management of Periprosthetic Joint Infection After Total Hip Arthroplasty Using a Custom Made Articulating Spacer (CUMARS); the Exeter Experience. *The Journal of Arthroplasty.*29(9):1813-1818.

6. Schairer WW, Sing DC, Vail TP, Bozic KJ. Causes and frequency of unplanned hospital readmission after total hip arthroplasty. *Clinical Orthopaedics & Related Research.* 2014;472(2):464-470.

7. Puhto AP, Puhto TM, Niinimaki TT, Leppilahti JI, Syrjala HP. Two-stage revision for prosthetic joint infection: outcome and role of reimplantation microbiology in 107 cases. *J Arthroplasty.* 2014;29(6):1101-1104.

8. Liu K, Zheng J, Jin Y, Zhao YQ. Application of temporarily functional antibiotic-containing bone cement prosthesis in revision hip arthroplasty. *European journal of orthopaedic surgery & traumatology : orthopedie traumatologie.* 2014;24(1):51-55.

9. Klatte TO, Kendoff D, Sabihi R, Kamath AF, Rueger JM, Gehrke T. Tantalum acetabular augments in one-stage exchange of infected total hip arthroplasty: a case-control study. *J Arthroplasty.* 2014;29(7):1443-1448.

10. Klatte TO, Kendoff D, Kamath AF, et al. Single-stage revision for fungal peri-prosthetic joint infection: a single-centre experience. *Bone Joint J.* 2014;96-B(4):492-496.

11. van Diemen MP, Colen S, Dalemans AA, Stuyck J, Mulier M. Two-stage revision of an infected total hip arthroplasty: a follow-up of 136 patients. *Hip international : the journal of clinical and experimental research on hip pathology and therapy.* 2013;23(5):445-450.

12. Spiegl U, Friederichs J, Patzold R, Militz M, Josten C, Buhren V. Risk factors for failed two-stage procedure after chronic posttraumatic periprosthetic hip infections. *Arch Orthop Trauma Surg.* 2013;133(3):421-428.

13. Morley JR, Blake SM, Hubble MJ, Timperley AJ, Gie GA, Howell JR. Preservation of the original femoral cement mantle during the management of infected cemented total hip replacement by two-stage revision. *Journal of Bone & Joint Surgery - British Volume.* 2012;94(3):322-327.

14. Leung F, Richards CJ, Garbuz DS, Masri BA, Duncan CP. Two-stage total hip arthroplasty: How often does it control methicillin-resistant infection? *Clin Orthop.* 2011;469(4):1009-1015.

15. Fleck EE, Spangehl MJ, Rapuri VR, Beauchamp CP. An articulating antibiotic spacer controls infection and improves pain and function in a degenerative septic hip. *Clinical Orthopaedics & Related Research.* 2011;469(11):3055-3064.

16. Abolghasemian M, Sternheim A, Shakib A, Safir OA, Backstein D. Is arthroplasty immediately after an infected case a risk factor for infection? *Clinical Orthopaedics & Related Research.* 2013;471(7):2253-2258.

17. Trojani C, d'Ollonne T, Saragaglia D, et al. One-stage bilateral total hip arthroplasty: functional outcomes and complications in 112 patients. *Orthop Traumatol Surg Res.* 2012;98(6 Suppl):S120-123.

18. Jiang Q, Xu ZH, Chen DY, et al. [Revision total hip arthroplasty using a cementless prosthesis]. *Zhonghua wai ke za zhi [Chinese journal of surgery].* 2012;50(5):393-397.

19. Lombardi AV, Jr., Berend KR, Adams JB. Partial two-stage exchange of the infected total hip replacement using disposable spacer moulds. *Bone Joint J.* 2014;96-B(11 Supple A):66-69.

20. Ekpo TE, Berend KR, Morris MJ, Adams JB, Lombardi AV, Jr. Partial two-stage exchange for infected total hip arthroplasty: a preliminary report. *Clinical Orthopaedics & Related Research.* 2014;472(2):437-448.

21. Barbaric K, Aljinovic A, Dubravcic ID, Delimar D, Bicanic G. Patient satisfaction after revision hip arthroplasty or resection hip arthroplasty due to periprosthetic infection. *Coll Antropol.* 2014;38(2):605-610.

22. Bedair H, Ting N, Bozic KJ, Della Valle CJ, Sporer SM. Treatment of early postoperative infections after THA: a decision analysis. *Clinical Orthopaedics & Related Research.* 2011;469(12):3477-3485.

23. Leonard HA, Liddle AD, Burke O, Murray DW, Pandit H. Single- or two-stage revision for infected total hip arthroplasty? A systematic review of the literature. *Clinical Orthopaedics & Related Research.* 2014;472(3):1036-1042.

24. Lange J, Troelsen A, Thomsen RW, Soballe K. Chronic infections in hip arthroplasties: comparing risk of reinfection following one-stage and two-stage revision: a systematic review and meta-analysis. *Clinical epidemiology.* 2012;4:57-73.

25. Tarasevicius S, Cebatorius A, Valaviciene R, Stucinskas J, Leonas L, Robertsson O. First outcome results after total knee and hip replacement from the Lithuanian arthroplasty register. *Medicina.* 2014;50(2):87-91.

26. Bansal A, Khatib ON, Zuckerman JD. Revision total joint arthroplasty: the epidemiology of 63,140 cases in New York State. *J Arthroplasty.* 2014;29(1):23-27.

27. Konan S, Soler A, Haddad FS. Revision hip replacement in patients 55 years of age and younger. *Hip International.* 2013;23(2):162-165.

28. Klatte TO, O'Loughlin PF, Citak M, Rueger JM, Gehrke T, Kendoff D. 1-stage primary arthroplasty of mechanically failed internally fixated of hip fractures with deep wound infection: good outcome in 16 cases. *Acta Orthop.* 2013;84(4):377-379.

29. Sabry FY, Szubski CR, Stefancin JJ, Klika AK, Higuera CA, Barsoum WK. Comparison of complications associated with commercially available and custom-made articulating spacers in two-stage total hip arthroplasty revision. *Current Orthopaedic Practice.* 2013;24(4):406-413.

30. Karam JA, Tokarski AT, Ciccotti M, Austin MS, Deirmengian GK. Revision total hip arthroplasty in younger patients: indications, reasons for failure, and survivorship. *The Physician and sportsmedicine.* 2012;40(4):96-101.

31. Wimmer MD, Randau TM, Petersdorf S, et al. Evaluation of an interdisciplinary therapy algorithm in patients with prosthetic joint infections. *Int Orthop.* 2013;37(11):2271-2278.

32. El Helou OC, Berbari EF, Lahr BD, et al. Management of prosthetic joint infection treated with two-stage exchange: the impact of antimicrobial therapy duration. *Current Orthopaedic Practice.* 2011;22(4):333-338.

33. Nelson CL, Jones RB, Wingert NC, Foltzer M, Bowen TR. Sonication of antibiotic spacers predicts failure during two-stage revision for prosthetic knee and hip infections. *Clin Orthop.* 2014;472(7):2208-2214.

34. Sorli L, Puig L, Torres-Claramunt R, et al. The relationship between microbiology results in the second of a two-stage exchange procedure using cement spacers and the outcome after revision total joint replacement for infection: the use of sonication to aid bacteriological analysis. *J Bone Joint Surg Br.* 2012;94(2):249-253.

35. Choi HR, von Knoch F, Kandil AO, Zurakowski D, Moore S, Malchau H. Retention treatment after periprosthetic total hip arthroplasty infection. *International Orthopaedics.* 2012;36(4):723-729.

**Appendix E.** Reference list of included studies

1. Buchholz H, Elson R, Engelbrecht E, Lodenkamper H, Rottger J, Siegel A. Management of deep infection of total hip replacement. *J Bone Joint Surg Am.* 1981;63-B(3):342-353.

2. Loty B, Postel M, Evrard J, et al. One stage revision of infected total hip replacements with replacement of bone loss by allografts. Study of 90 cases of which 46 used bone allografts. *Int Orthop.* 1992;16(4):330-338.

3. Miley GB, Scheller AD, Turner RH. Medical and surgical treatment of the septic hip with one-stage revision arthroplasty. *Clin Orthop.* 1982;170(76-82).

4. Mulcahy DM, O'Byrne JM, Fenelon GE. One stage surgical management of deep infection of total hip arthroplasty. *Irish J Med Sci.* 1996;165(1):17-19.

5. Raut VV, Siney PD, Wroblewski BM. One-stage revision of total hip arthroplasty for deep infection. Long-term followup. *Clin Orthop.* 1995;321:202-207.

6. Wroblewski BM. One-stage revision of infected cemented total hip arthroplasty. *Clin Orthop.* 1986;211:103-107.

7. Schneider R. The infected total prosthesis. *Orthopade.* 1989;18(6):527-532.

8. Winkler H, Kaudela K, Stoiber A, Menschik F. Bone grafts impregnated with antibiotics as a tool for treating infected implants in orthopedic surgery - One stage revision results. *Cell and Tissue Banking.* 2006;7 (4):319-323.

9. Rudelli S, Uip D, Honda E, Lima AL. One-stage revision of infected total hip arthroplasty with bone graft. *J Arthroplasty.* 2008;23(8):1165-1177.

10. Ure KJ, Amstutz HC, S N, Schmalzried TP. Direct-exchange arthroplasty for the treatment of infection after total hip replacement. An average ten-year follow-up. *J Bone Joint Surg Am.* 1998;80-A(7):961-968.

11. Wagner M. Local antisepsis in revision surgery of infected total hip prostheses. *Orthopade.* 1995;24(4):319-325.

12. Wagner M, Willenegger H. Local antisepsis in revision of infected total hip replacement. *Acta Chir Austriaca.* 1997;29 (Suppl 133):64-68.

13. Sofer D, Regenbrecht B, Pfeil J. Early results of one-stage septic revision arthroplasties with antibiotic-laden cement. A clinical and statistical analysis. *Orthopade.* 2005;34(6):592-602.

14. Elson RA. Exchange arthroplasty for infection. Perspectives from the United Kingdom. *Orthop Clin North Am.* 1993;24(4):761-767.

15. Vielpeau C, Lortat-Jacob A. Management of the infected hip prostheses. *Rev Chir Orthop Reparatrice Appar Mot.* 2002;88(Suppl 1):159-216.

16. Sanzen L, Carlsson A, Josefsson G, Lindberg LT. Revision operations on infected total hip arthroplasties. *Clin Orthop.* 1988;229:165-172.

17. Carlsson A, Josefsson G, Lindberg L. Revision with gentamicin-impregnated cement for deep infections in total hip arthroplasties. *J Bone Joint Surg Am.* 1978;60-A(8):1059-1064.

18. Hope PG, Kristinsson KG, Norman P, Elson RA. Deep infection of cemented total hip arthroplasties caused by coagulase-negative staphylococci. *J Bone Joint Surg Am.* 1989;71-B(5):851-855.

19. Schneider R. The infected total hip replacement prosthesis. *Helvetica Chirurgica Acta.* 1978;45(4-5):553-566.

20. Callaghan JJ, Katz RP, Johnston RC. One-stage revision surgery of the infected hip. A minimum 10-year followup study. *Clin Orthop.* 1999;369:139-143.

21. De Man FH, Sendi P, Zimmerli W, Maurer TB, Ochsner PE, Ilchmann T. Infectiological, functional, and radiographic outcome after revision for prosthetic hip infection according to a strict algorithm. *Acta Orthop.* 2011;82(1):27-34.

22. Katz RP, Callaghan JJ, Johnston RC. A minimum ten year follow-up study of one stage reimplantation of the infected total hip. *Orthop Trans.* 1994;18:993.

23. Ketterl R, Henly MB, Stübinger B, Beckurts T, Claudi B. Analysis of three operative techniques for infected total hip replacements. *Orthop Trans.* 1988;12:715.

24. Collin P, Siret P, Lahogue J-F, Lambotte J-C, Thomazeau H, Langlais F. Infected hip prosthesis. One- or two-stage replacement? Comparison of 2 series. *Ann Orthop Ouest.* 2002;34:129-134.

25. Giulieri SG, Graber P, Ochsner PE, Zimmerli W. Management of infection associated with total hip arthroplasty according to a treatment algorithm. *Infection.* 2004;32(4):222-228.

26. Lecuire F, Collodel M, Basso M, Rubini J, Gontier D, Carrere J. Revision of infected total hip prostheses by ablation reimplantation of an uncemented prosthesis. 57 case reports. *Rev Chir Orthop Reparatrice Appar Mot.* 1999;85(4):337-348.

27. Garcia S, Soriano A, Esteban P, Almela M, Gallart X, Mensa J. Usefulness of adding antibiotic to cement in one stage exchange of chronic infection in total hip arthroplasty. *Medicina Clinica.* 2005;125(4):138-139.

28. Wilson PD, Aglietti P, Salvati EA. Subacute sepsis of the hip treated by antibiotics and cemented prosthesis. *J Bone Joint Surg Am.* 1974;56-A(5):879-898.

29. Salvati EA, Chekofsky KM, Brause BD, Wilson PD. Reimplantation in infection: a 12-year experience. *Clin Orthop.* 1982;170:62-75.

30. Hughes PW, Salvati EA, Wilson PD, Blumenfeld EL. Treatment of Subacute Sepsis of the Hip by Antibiotics and Joint Replacement Criteria For Diagnosis With Evaluation of Twenty-Six Cases. *Clin Orthop.* 1979;141:143-157.

31. Wu CC, Chen WJ. One-stage revision surgery to treat hip infected nonunion after stabilization with a sliding compression screw. *Arch Orthop Trauma Surg.* 2003;123(8):383-387.

32. Yoo JJ, Kwon YS, Koo KH, Yoon KS, Kim YM, Kim HJ. One-stage cementless revision arthroplasty for infected hip replacements. *Int Orthop.* 2009;33(5):1195-1201.

33. Oussedik SI, Dodd MB, Haddad FS. Outcomes of revision total hip replacement for infection after grading according to a standard protocol. *J Bone Joint Surg Am.* 2010;92-B(9):1222-1226.

34. Gao H, Lv H. One-stage revision operations for infection after hip arthroplasty. *Chinese Journal of Reparative & Reconstructive Surgery.* 2008;22(1):5-8.

35. Garvin KL, Evans BG, Salvati EA, Brause BD. Palacos gentamicin for the treatment of deep periprosthetic hip infections. *Clin Orthop.* 1994;298:97-105.

36. Jenny JY, Lengert R, Diesinger Y, Gaudias J, Boeri C, Kempf JF. Routine one-stage exchange for chronic infection after total hip replacement. *Int Orthop.* 2014;38(12):2477-2481.

37. Zeller V, Lhotellier L, Marmor S, et al. One-stage exchange arthroplasty for chronic periprosthetic hip infection: results of a large prospective cohort study. *Journal of Bone & Joint Surgery - American Volume.* 2014;96(1):e1.

38. Wolf M, Clar H, Friesenbichler J, et al. Prosthetic joint infection following total hip replacement: results of one-stage versus two-stage exchange. *Int Orthop.* 2014;38(7):1363-1368.

39. Bori G, Munoz-Mahamud E, Cune J, Gallart X, Fuster D, Soriano A. One-stage revision arthroplasty using cementless stem for infected hip arthroplasties. *Journal of Arthroplasty.* 2014;29(5):1076-1081.

40. Choi HR, Kwon YM, Freiberg AA, Malchau H. Comparison of one-stage revision with antibiotic cement versus two-stage revision results for infected total hip arthroplasty. *Journal of Arthroplasty.* 2013;28(8 Suppl):66-70.

41. Klouche S, Leonard P, Zeller V, et al. Infected total hip arthroplasty revision: one- or two-stage procedure? *Orthop Traumatol Surg Res.* 2012;98(2):144-150.

42. Engesaeter LB, Dale H, Schrama JC, Hallan G, Lie SA. Surgical procedures in the treatment of 784 infected THAs reported to the Norwegian Arthroplasty Register. *Acta Orthopaedica.* 2011;82(5):530-537.

43. Toulson C, Walcott-Sapp S, Hur J, et al. Treatment of infected total hip arthroplasty with a 2-stage reimplantation protocol: update on "our institution's" experience from 1989 to 2003. *J Arthroplasty.* 2009;24(7):1051-1060.

44. Fitzgerald RH, Jones DR. Hip implant infection: Treatment with resection arthroplasty and late total hip arthroplasty. *Am J Med.* 1985;78(6):225-228.

45. Stockley I, Mockford BJ, Hoad-Reddick A, Norman P. The use of two-stage exchange arthroplasty with depot antibiotics in the absence of long-term antibiotic therapy in infected total hip replacement. *J Bone Joint Surg Am.* 2008;90-B(2):145-148.

46. Hsieh PH, Huang KC, Lee PC, Lee MS. Two-stage revision of infected hip arthroplasty using an antibiotic-loaded spacer: Retrospective comparison between short-term and prolonged antibiotic therapy. *J Antimicrob Chemother.* 2009;64(2):392-397.

47. Chen WS, Fu TH, Wang JW. Two-stage reimplantation of infected hip arthroplasties. *Chang Gung Medical Journal.* 2009;32(2):188-197.

48. Haddad FS, Muirhead-Allwood SK, Manktelow AR, Bacarese-Hamilton I. Two-stage uncemented revision hip arthroplasty for infection. *J Bone Joint Surg Am.* 2000;82-B(5):689-694.

49. Lieberman JR, Callaway GH, Salvati EA, Pellicci PM, Brause BD. Treatment of the infected total hip arthroplasty with a two-stage reimplantation protocol. *Clin Orthop.* 1994;301:205-212.

50. Whittaker JP, Warren RE, Jones RS, Gregson PA. Is prolonged systemic antibiotic treatment essential in two-stage revision hip replacement for chronic Gram-positive infection (Journal of Bone and Joint Surgery - Series B (2009) 91-B, (44-51)). *Journal of Bone and Joint Surgery - Series B.* 2009;91 (5):700.

51. Colyer RA, Capello WN. Surgical treatment of the infected hip implant. Two-stage reimplantation with a one-month interval. *Clin Orthop.* 1994;298:75-79.

52. Fink B, Grossmann A, Fuerst M, Schafer P, Frommelt L. Two-stage cementless revision of infected hip endoprostheses. *Clin Orthop.* 2009;467(7):1848-1858.

53. Cabrita HB, Croci AT, Camargo OP, Lima AL. Prospective study of the treatment of infected hip arthroplasties with or without the use of an antibiotic-loaded cement spacer. *Clinics.* 2007;62(2):99-108.

54. Cordero-Ampuero J, Esteban J, Garcia-Cimbrelo E. Oral antibiotics are effective for highly resistant hip arthroplasty infections. *Clin Orthop.* 2009;467(9):2335-2342.

55. McKenna PB, O'Shea K, Masterson EL. Two-stage revision of infected hip arthroplasty using a shortened post-operative course of antibiotics. *Arch Orthop Trauma Surg.* 2009;129(4):489-494.

56. Piriou P, de Loynes B, Garreau de Loubresse C, Judet T. Use of combined gallium-technetium scintigraphy to determine the interval before second-stage prosthetic reimplantation in hip arthroplasty infection: a consecutive series of 30 cases. *Rev Chir Orthop Reparatrice Appar Mot.* 2003;89(4):287-296.

57. Fehring TK, Calton TF, Griffin WL. Cementless fixation in 2-stage reimplantation for periprosthetic sepsis. *J Arthroplasty.* 1999;14(2):175-181.

58. Evans RP. Successful treatment of total hip and knee infection with articulating antibiotic components: a modified treatment method. *Clin Orthop.* 2004;427:37-46.

59. Wilson MG, Dorr LD. Reimplantation of infected total hip arthroplasties in the absence of antibiotic cement. *J Arthroplasty.* 1989;4(3):263-269.

60. Yamamoto K, Miyagawa N, Masaoka T, Katori Y, Shishido T, Imakiire A. Clinical effectiveness of antibiotic-impregnated cement spacers for the treatment of infected implants of the hip joint. *J Orthop Sci.* 2003;8(6):823-828.

61. Cordero-Ampuero J, Esteban J, Garcia-Cimbrelo E, Munuera L, Escobar R. Low relapse with oral antibiotics and two-stage exchange for late arthroplasty infections in 40 patients after 2-9 years. *Acta Orthop.* 2007;78(4):511-519.

62. Nelson CL, Evans RP, Blaha JD, Calhoun J, Henry SL, Patzakis MJ. A comparison of gentamicin-impregnated polymethylmethacrylate bead implantation to conventional parenteral antibiotic therapy in infected total hip and knee arthroplasty. *Clin Orthop.* 1993;295:96-101.

63. Magnan B, Regis D, Biscaglia R, Bartolozzi P. Preformed acrylic bone cement spacer loaded with antibiotics: use of two-stage procedure in 10 patients because of infected hips after total replacement. *Acta Orthop Scand.* 2001;72(6):591-594.

64. Ladero Morales F, Fernandez Gonzalez J, Blanco Ortiz F, Martinez Martin J, Garcia Araujo C. Treatment of infected hip arthroplasty. Retrospective study. *Revista de Ortopedia y Traumatologia.* 1999;43(2):84-92.

65. Antti-Poika I, Santavirta S, Konttinen YT, Honkanen V. Outcome of the infected hip arthroplasty. A retrospective study of 36 patients. *Acta Orthop Scand.* 1989;60(6):670-675.

66. Ritter MA, Farris A. Outcome of infected total joint replacement. *Orthopedics.* 2010;33(3).

67. Wang Y, Hao L, Zhou Y, et al. Clinical experience of treating infection after total hip arthroplasty. *Chinese Journal of Surgery.* 2005;43(20):1313-1316.

68. Weber E, Cometta A, Blanc CH, Leyvraz PF. Review of infected total arthroplasties of the hip and knee-apropos of 28 cases. *Swiss Surgery.* 2000;6(6):335-342.

69. Shen B, Huang Q, Yang J, Zhou ZK, Kang PD, Pei FX. Extensively coated non-modular stem used in two-stage revision for infected total hip arthroplasty: mid-term to long-term follow-up. *Orthopaedic surgery.* 2014;6(2):103-109.

70. Schwarzkopf R, Mikhael B, Wright E, Estok DM, 2nd, Katz JN. Treatment failure among infected periprosthetic total hip arthroplasty patients. *The open orthopaedics journal.* 2014;8:118-124.

71. Cabo J, Euba G, Saborido A, et al. Clinical outcome and microbiological findings using antibiotic-loaded spacers in two-stage revision of prosthetic joint infections. *J Infect.* 2011;63(1):23-31.

72. Johnson AJ, Zywiel MG, Jones LC, Delanois RE, Stroh DA, Mont MA. Reduced re-infection rates with postoperative oral antibiotics after two-stage revision hip arthroplasty. *BMC musculoskeletal disorders.* 2013;14:123.

73. Shen H, Wang QJ, Zhang XL, et al. [Cementless two-staged total hip arthroplasty for chronic periprosthetic infection]. *Chung Hua Wai Ko Tsa Chih.* 2012;50(5):402-406.

74. Neumann DR, Hofstaedter T, List C, Dorn U. Two-stage cementless revision of late total hip arthroplasty infection using a premanufactured spacer. *Journal of Arthroplasty.* 2012;27(7):1397-1401.

75. Macheras GA, Koutsostathis SD, Kateros K, Papadakis S, Anastasopoulos P. A two stage re-implantation protocol for the treatment of deep periprosthetic hip infection. Mid to long-term results. *Hip International.* 2012;22 Suppl 8:S54-61.

76. Zou YG, Feng ZQ, Xing JS, Peng ZH, Luo X. [Two-stage revision for treatment of periprosthetic infection following hip arthroplasty]. *Nan fang yi ke da xue xue bao = Journal of Southern Medical University.* 2011;31(4):690-693.

77. Wang L, Hu Y, Dai Z, Zhou J, Li M, Li K. [Mid-term effectiveness of two-stage hip prosthesis revision in treatment of infection after hip arthroplasty]. *Chung Kuo Hsiu Fu Chung Chien Wai Ko Tsa Chih.* 2011;25(6):646-649.

78. Pattyn C, De Geest T, Ackerman P, Audenaert E. Preformed gentamicin spacers in two-stage revision hip arthroplasty: functional results and complications. *International Orthopaedics.* 2011;35(10):1471-1476.

79. Romano CL, Romano D, Albisetti A, Meani E. Preformed antibiotic-loaded cement spacers for two-stage revision of infected total hip arthroplasty. Long-term results. *Hip International.* 2012;22 Suppl 8:S46-53.

80. Uchiyama K, Takahira N, Fukushima K, et al. Two-stage revision total hip arthroplasty for periprosthetic infections using antibiotic-impregnated cement spacers of various types and materials. *ScientificWorldJournal.* 2013;2013:147248.

81. Berend KR, Lombardi AV, Jr., Morris MJ, Bergeson AG, Adams JB, Sneller MA. Two-stage treatment of hip periprosthetic joint infection is associated with a high rate of infection control but high mortality. *Clinical Orthopaedics & Related Research.* 2013;471(2):510-518.

82. Borowski M, Kusz D, Wojciechowski P, Cielinski L. Treatment for periprosthetic infection with two-stage revision arthroplasty with a gentamicin loaded spacer. The clinical outcomes. *Ortop.* 2012;14(1):41-54.

83. D'Angelo F, Negri L, Binda T, Zatti G, Cherubino P. The use of a preformed spacer in two-stage revision of infected hip arthroplasties. *Musculoskelet Surg.* 2011;95(2):115-120.

84. Lim SJ, Moon YW, Park YS. Is extended trochanteric osteotomy safe for use in 2-stage revision of periprosthetic hip infection? *J Arthroplasty.* 2011;26(7):1067-1071.

85. Ibrahim MS, Raja S, Khan MA, Haddad FS. A multidisciplinary team approach to two-stage revision for the infected hip replacement: a minimum five-year follow-up study. *Bone Joint J.* 2014;96-B(10):1312-1318.

**Table A.** Characteristics of studies included in review

| **Lead Author, Publication Date (Reference No.)** | **Location** | **Year of study** | **Mean /median age (years)** | **% male** | **Follow up**  **Mean/median (months)** | **Type of re-implantation** | **Use of spacer** | **No. of re-infections** | **No. of participants** | **Quality score** |
| --- | --- | --- | --- | --- | --- | --- | --- | --- | --- | --- |
| **One-stage** |  |  |  |  |  |  |  |  |  |  |
| Bucholz, 1981^1^ | Germany | 1968–1977 | 58.8 | 39.7 | 52.0 | Cemented | NA | 99 | 640 | 10 |
| Loty, 1992^2^ | France | 1980–1988 | 65.7 | NS | 47.0 | Cemented | NA | 8 | 90 | 13 |
| Miley, 1982^3^ | USA | 1969-1979 | 57.7 | 53.0 | 48.5 | Cemented | NA | 8 | 101 | 9 |
| Mulcahy, 1996^4^ | Ireland | NS | 64.0 | 68.0 | 53.0 | Cemented | NA | 0 | 15 | 14 |
| Raut, 1995^5^; Wroblewski, 1986^6^ | UK | 1979-1990 | 64.5 | 48.0 | 93 | Cemented | NA | 6 | 183 | 11 |
| Schneider, 1989^7^ | Switzerland | 1973-1988 | NS | NS | NS | NS | NA | 19 | 72 | 11 |
| Winkler, 2006^8^ | Austria | 1998-2004 | NS | NS | 38.4 | Cementless | NA | 3 | 37 | 12 |
| Rudelli, 2008^9^ | Brazil | 1989-2000 | 61.3 | 41.0 | 103.0 | Cementless | NA | 0 | 32 | 12 |
| Ure, 1998^10^ | USA | 1979-1990 | 61.4 | 80.0 | 118.0 | Cemented | NA | 0 | 20 | 15 |
| Wagner, 1995^11^; Wagner, 1997^12^ | Germany | 1991-1993 | 64.6 | NS | NS | NS | NA | 4 | 18 | 11 |
| Sofer, 2005^13^ | Germany | NS | NS | NS | 17.6 | Cemented | NA | 1 | 17 | 12 |
| Elson, 1993^14^ | UK | NS | NS | NS | NS | NS | NA | 33 | 235 | 11 |
| Vielpeau, 2002^15^ | France | 1998 | NS | NS | 36.0 | NS | NA | 15 | 127 | 11 |
| Sanzen, 1988^16^; Carlsson, 1978^17^ | Sweden | 1974-1981 | 64.0 | 53.0 | 71.0 | Cemented | NA | 17 | 78 | 15 |
| Hope, 1989^18^ | UK | NS | 64.0 | 44.0 | 45.0 | Cemented | NA | 9 | 72 | 11 |
| Schneider, 1978^19^ | Germany | NS | NS | NS | NS | NS | NA | 5 | 30 | 11 |
| Callaghan, 1999^20^ | USA | 1977-1983 | 65.3 | 50.0 | 109.2 | Cemented | NA | 2 | 24 | 15 |
| De Man, 2011^21^ | Switzerland | 1985-2004 | 70.0 | 57.0 | 45.6 | NS | NA | 1 | 24 | 10 |
| Katz, 1994^22^ | USA | NS | NS | NS | NS | NS | NS | 2 | 24 | 12 |
| Ketterl, 1988^23^ | Germany | 1976-1986 | 69.0 | 42.0 | 32.0 | NS | NA | 7 | 21 | 12 |
| Collin, 2002^24^ | France | 1992-1999 | 63.3 | NS | 26.0 | NS | NA | 1 | 17 | 13 |
| Giulieri, 2004^25^ | Switzerland | 1985-2001 | 72.0 | 67.0 | 28.0 | NS | NA | 1 | 16 | 13 |
| Lecuire, 1999^26^ | France | 1982-1997 | 70.6 | NS | 79.2 | Cementless | NA | 1 | 16 | 11 |
| Garcia, 2005^27^ | Spain | NS | NS | NS | NS | NS | NA | 0 | 14 | 11 |
| Wilson, 1974^28^; Salvati, 1982^29^ | USA | 1968-1971 | 63.0 | 21.0 | 36.0 | Cemented | NA | 2 | 14 | 14 |
| Hughes, 1979^30^ | USA | 1971-1975 | 62.0 | 38.0 | 51.0 | NS | NA | 1 | 13 | 13 |
| Wu, 2003^31^ | Taiwan | NS | NS | NS | NS | NS | NA | 1 | 13 | 15 |
| Yoo, 2009^32^ | South Korea | 1991-2005 | 50.0 | 67.0 | 86.4 | Cementless | NA | 1 | 12 | 14 |
| Oussedik, 2010^33^ | UK | 1999-2002 | 65.0 | 42.0 | 81.6 | Cemented | NA | 0 | 11 | 12 |
| Gao, 2008^34^ | China | 1999-2005 | 63.0 | 67.0 | 19.0 | NS | NA | 0 | 10 | 12 |
| Garvin, 1994^35^ | USA | 1983-1986 | 66.9 | 45.0 | 68.4 | NS | NA | 1 | 10 | 12 |
| Jenny, 2014^36^ | France | 2007-2010 | 73.0 | 63.0 | 37.0 | Cemented | NA | 6 | 65 | 15 |
| Zeller, 2014^37^ | France | 2002-2010 | 71.0 | 58.0 | 41.6 | Cementless | NA | 6 | 157 | 16 |
| Wolf, 2014^38^ | Austria | 1985-2004 | 67.0 | 48.6 | 24.0 | Cementless | NA | 4 | 37 | 14 |
| Bori, 2014^39^ | Spain | 1998-2007 | 72.4 | 37.5 | 44.6 | Cementless | NA | 1 | 24 | 14 |
| Choi, 2013^40^ | USA | 1999-2009 | 65.0** | 52.9 | 62.0 | Cemented | NA | 3 | 17 | 15 |
| Klouche, 2012^41^ | France | 2002-2006 | 63.6 | 52.6 | 35.0 | Cemented | NA | 0 | 38 | 15 |
| Engesaeter, 2011^42^ | Norway | 1987-2009 | 72.0 | 55.0 | 24.0*** | NS | NA | 22 | 192 | 13 |
| **Two-stage** |  |  |  |  |  |  |  |  |  |  |
| Toulson, 2009^43^ | USA | 1989-2003 | 54.7 | 59.0 | 64.8 | Cemented | Spacer | 11 | 132 | 12 |
| Fitzgerald, 1985^44^ | USA | 1969-1979 | 61.0 | 50.0 | 49.0 | NS | NS | 11 | 131 | 11 |
| Stockley, 2008^45^ | UK | 1991-2004 | 64.0 | 55.0 | 74.0 | Cemented | Beads | 9 | 114 | 12 |
| Hsieh, 2009^46^ | Taiwan | 2002-2005 | 61.0 | 61.0 | 43.0 | Cemented | Spacer | 8 | 99 | 13 |
| Chen, 2009^47^ | Taiwan | 1993-2005 | 51.5 | 72.0 | 67.2 | Both | Beads | 7 | 57 | 12 |
| Haddad, 2000^48^ | UK | 1988-1992 | 60.0 | 46.0 | 69.6 | Cementless | Beads | 4 | 50 | 13 |
| Lieberman, 1994^49^ | USA | 1985-1988 | 69.0 | 47.0 | 40.0 | Cemented | NS | 8 | 47 | 14 |
| Whittaker, 2009^50^ | UK | 1998-2003 | 69.0 | 49.0 | 49.0 | Both | Spacer | 6 | 43 | 15 |
| Colyer, 1994^51^ | USA | NS | 57.0 | 44.0 | 36.0 | Cementless | No spacer | 5 | 41 | 14 |
| Fink, 2009^52^ | Germany | 2002-2006 | 69.0 | 44.0 | 35.0 | Cementless | Spacer | 0 | 40 | 13 |
| Cabrita, 2007a^53^ | Brazil | 1996-2003 | 54.6 | 58.0 | 48.0 | NS | Spacer | 4 | 38 | 15 |
| Cabrita, 2007b^53^ | Brazil | 1996-2003 | 54.6 | 58.0 | 48.0 | NS | No spacer | 10 | 30 | 15 |
| Cordero-Ampuero, 2009^54^ | Spain | 1997-2007 | 71.8 | 36.0 | 52.8 | Cemented | No spacer | 3 | 36 | 13 |
| McKenna, 2009^55^ | Ireland | 2001-2004 | 63.0 | 57.0 | 35.0 | Cementless | Spacer | 3 | 31 | 14 |
| Piriou, 2003^56^ | France | 1987-1997 | 64.0 | 57.0 | 60.0 | NS | No spacer | 3 | 30 | 13 |
| Fehring, 1999^57^ | USA | NS | NS | NS | 41.0 | Cementless | Beads | 1 | 25 | 14 |
| Evans, 2004^58^ | USA | 1995-2002 | 65.0 | 55.0 | 24.0 | Cemented | Spacer | 4 | 23 | 15 |
| Wilson, 1989^59^ | USA | NS | 56.0 | NS | 48.0 | Both | NS | 2 | 22 | 14 |
| Yamamoto, 2003^60^ | Japan | 1998-2002 | 61.8 | 35.0 | 38.0 | Cementless | Spacer | 0 | 17 | 14 |
| Cordero-Ampuero, 2007^61^ | Spain | 1996-2003 | 72.0 | 25.0 | 48.0 | Cemented | No spacer | 1 | 16 | 15 |
| Nelson, 1993a^62^ | USA | 1985-1990 | 60.0 | 68.0 | 32.0 | Cemented | Beads | 2 | 12 | 13 |
| Nelson, 1993b^62^ | USA | 1985-1990 | 60.0 | 68.0 | 32.0 | Cemented | No spacer | 4 | 10 | 13 |
| Magnan, 2001^63^ | Italy | 1996-1999 | 72.0 | 70.0 | 35.0 | Cemented | Spacer | 2 | 10 | 13 |
| Vielpeau, 2002^15^ | France | 1998 | NS | NS | 36.0 | NS | NS | 33 | 222 | 11 |
| Ketterl, 1988^23^ | Germany | 1976-1986 | 69.0 | 42.0 | 32.0 | NS | NS | 17 | 161 | 12 |
| Elson, 1993^14^ | UK | NS | NS | NS | NS | NS | NS | 3 | 61 | 11 |
| De Man, 2011^21^ | Switzerland | 1985-2004 | 70.0 | 57.0 | 45.6 | NS | NS | 1 | 55 | 10 |
| Lecuire, 1999^26^ | France | 1982-1997 | 70.6 | NS | 79.2 | Cementless | NS | 1 | 41 | 11 |
| Oussedik, 2010^33^ | UK | 1999-2002 | 65.0 | NS | 81.6 | Cemented | Spacer | 2 | 39 | 12 |
| Ladero Morales, 1999^64^ | Spain | 1985-1995 | 74.0 | 53.0 | 57.6 | NS | NS | 3 | 37 | 12 |
| Sanzen, 1988^16^; Carlsson, 1978^17^ | Sweden | 1974-1981 | 64.0 | 53.0 | 71.0 | Cemented | Beads | 8 | 32 | 15 |
| Giulieri, 2004^25^ | Switzerland | 1984-2001 | 72.0 | 67.0 | 28.0 | NS | NS | 3 | 31 | 13 |
| Garvin, 1994^35^ | USA | 1983-1986 | 66.9 | 45.0 | 68.4 | Cemented | NS | 1 | 30 | 12 |
| Antti-Poika, 1989^65^ | Finland | 1976-1985 | 57.0 | 39.0 | 72.0 | Cemented | NS | 5 | 26 | 12 |
| Ritter, 2010^66^ | USA | 1969-2004 | 66.2 | 52.0 | 82.8 | NS | NS | 5 | 17 | 12 |
| Collin, 2002^24^ | France | 1992-1999 | 63.3 | 50.0 | 26.0 | NS | NS | 1 | 15 | 13 |
| Wang, 2005^67^ | China | 1975-2004 | 54.0 | 42.0 | 38.4 | NS | NS | 0 | 15 | 12 |
| Weber, 2000^68^ | Switzerland | 1990-1994 | 73.0 | 43.0 | 46.0 | NS | NS | 0 | 14 | 12 |
| Hughes, 1979^30^ | USA | 1971-1975 | 62.0 | 38.0 | 51.0 | NS | NS | 1 | 13 | 13 |
| Wolf, 2014^38^ | Austria | 1985-2004 | 60.4 | 52.7 | 24.0 | Cementless | Spacer | 5 | 55 | 14 |
| Shen, 2014^69^ | China | 2005-2006 | 65.0 | 60.6 | 72.0 | Cementless | Spacer | 0 | 33 | 14 |
| Schwarzkopf, 2014^70^ | USA | 2001-2011 | 62.3 | NS | 32.4 | Cemented | Spacer | 3 | 62 | 12 |
| Cabo, 2011^71^ | Spain | 2004-2009 | 68.0 | 42.0 | 24.0 | Cementless | Spacer | 1 | 19 | 15 |
| Johnson, 2013^72^ | USA | 2000-2007 | 58.0 | 43.9 | 45 | NS | Spacer | 6 | 66 | 14 |
| Choi, 2013^40^ | USA | 1999-2009 | 65.0** | 53.0 | 70.0 | Cemented | Spacer | 11 | 44 | 14 |
| Shen, 2012^73^ | China | NS | 64.0 | 39.1 | 51.6 | Cementless | Spacer | 2 | 23 | 12 |
| Neumann, 2012^74^ | Austria | 2000-2008 | 25-84† | 56.8 | 67.0 | Cementless | Spacer | 1 | 44 | 13 |
| Macheras, 2012^75^ | Greece | 1998-2004 | 67.0 | 34.3 | 139.2 | Both | No spacer | 2 | 35 | 13 |
| Klouche, 2012^41^ | France | 2002-2006 | 66.9 | 56.5 | 35.0 | Cemented | Spacer | 4 | 46 | 15 |
| Zou, 2011^76^ | China | 2006-2010 | NS | NS | 25.0 | Both | Spacer | 0 | 15 | 14 |
| Wang, 2011^77^ | China | 2002-2006 | 59.8 | 41.7 | 64.8 | NS | Spacer | 0 | 12 | 14 |
| Pattyn, 2011^78^ | Belgium | 2003-2009 | 65.4 | 49.2 | 36.0 | NS | Spacer | 2 | 61 | 16 |
| Engesaeter, 2011^42^ | Norway | 1987-2009 | 71.0 | 48.0 | 24.0*** | NS | NS | 15 | 283 | 13 |
| Romano, 2012^79^ | Italy | 2000-2010 | 60.3 | 33.3 | 60.0 | Cementless | Spacer | 10 | 183 | 13 |
| Uchiyama, 2013^80^ | Japan | 2000-2012 | 62.4 | 52.8 | 48.6 | Cementless | Spacer | 13 | 37 | 15 |
| Berend, 2013^81^ | USA | 1996-2009 | 65.0 | 53.0 | 54.0 | Cementless | Spacer | 32 | 202 | 12 |
| Borowski, 2012^82^ | Poland | 2007-2010 | NS | NS | > 6.0 | NS | Spacer | 3 | 29 | 12 |
| D’Angelo, 2011^83^ | Italy | 1999-2008 | 71.4 | 53.6 | 53.0 | Both | Spacer | 1 | 28 | 14 |
| Lim, 2011^84^ | South Korea | 2000-2007 | 58.5 | 47.8 | 63.0 | Cementless | Spacer | 1 | 23 | 14 |
| Ibrahim, 2014^85^ | UK | 2000-2008 | 68.0 | 40.8 | 103.2 | NS | Spacer | 5 | 125 | 15 |

NA, not applicable; NS, not stated; *, are number of hips; **, mean age for participants who were followed up; ***, results based on a 2-year survival analysis; †, age range; Both, some participants had cemented re-implantation and others cementless

**Figure A**. Rates of re-infection in unselected patients treated by one-stage revision, grouped according to study and population level characteristics

CI, confidence interval (bars); *, *P*-value for meta-regression; †, number of infections and participants do not add up to the grand total because of missing data

**Figure B**. Rates of re-infection in unselected patients treated by two-stage revision, grouped according to study and population level characteristics

CI, confidence interval (bars); *, *P*-value for meta-regression; †, number of infections and participants do not add up to the grand total because of missing data
